# Lentinan enhances CAR-T cell potency in solid tumors by optimizing T cell differentiation

**DOI:** 10.3389/fimmu.2025.1605488

**Published:** 2025-07-18

**Authors:** Xiangyun Niu, Pengchao Zhang, Zhongming Liu, Yexiao Tang, Shu Xu, Xiaochun Wan, Zhiming Xu, Guizhong Zhang

**Affiliations:** ^1^ Guangdong Immune Cell Therapy Engineering and Technology Research Center, Center for Protein and Cell-based Drugs, Institute of Biomedicine and Biotechnology, Shenzhen Institutes of Advanced Technology, Chinese Academy of Sciences, Shenzhen, China; ^2^ University of Chinese Academy of Sciences, Beijing, China; ^3^ Cancer Center, Shenzhen Guangming District People’s Hospital, Shenzhen, China

**Keywords:** CAR-T cell therapy, lentinan, central memory T cells, solid tumor, tumor microenvironment

## Abstract

CAR-T cell therapy has demonstrated remarkable success in treating hematologic malignancies; however, its efficacy in solid tumors remains constrained. In this study, we demonstrate that Lentinan (LTN), an active polysaccharide derived from *Lentinula edodes*, potently enhances CAR-T cell function against solid tumors. *In vitro*, LTN significantly augments CAR-T cell cytotoxicity and pro-inflammatory cytokine secretion (IL-2, IFN-γ). Mechanistically, LTN drives CAR-T cell differentiation into a memory phenotype, characterized by increased frequencies of CD44^+^CD62L^+^ central memory cells and enrichment of CD44^+^CD62L^+^TCF1^+^ stem-like memory cells, while concomitantly mitigating exhaustion, as evidenced by reduced surface expression of the checkpoint receptor TIM-3 and the exhaustion-associated marker CD317. These phenotypic and functional improvements correlate with LTN-mediated transcriptional upregulation of memory-associated factors *Tcf7* (encoding TCF1) and *Foxo1*. *In vivo*, the combination of LTN and CAR-T significantly enhances tumor control in syngeneic murine models of colon carcinoma and melanoma. This superior efficacy stems from enhanced CAR-T cell persistence, sustained intratumoral effector function, and reprogramming of tumor-associated macrophages (TAMs) toward an immunostimulatory M1-like phenotype. This work establishes LTN as a clinically actionable immunomodulator that synergizes with CAR-T cells by intrinsically enhancing their fitness and persistence while extrinsically remodeling the suppressive tumor microenvironment. It provides a novel, translatable strategy to potentiate CAR-T therapy against solid tumors.

## 1 Introduction

Chimeric antigen receptor (CAR) T cell therapy is an adoptive cell therapy (ACT) in which autologous T cells are genetically engineered to express CAR to specifically kill tumor cells (1). CAR-T cell therapy is an opportunity to treat patients who have not responded to other first-line cancer therapies, and has shown superior antitumor effects in the treatment of hematologic malignancies (2). Although CAR-T cell therapy has achieved remarkable clinical success in treating B cell malignancies, its efficacy against solid tumors remains suboptimal (3). This disparity can be attributed to the distinct characteristics of solid tumors, including the immunosuppressive tumor microenvironment (TME), limited CAR-T cell infiltration, and tumor antigen heterogeneity. These factors collectively hinder the therapeutic potential of CAR-T cells in solid tumor contexts (4, 5).

To address the challenges associated with CAR-T cell therapy in solid tumors, various strategies have been developed. These include multi-targeted CAR strategies, enhanced trafficking and infiltration of CAR-T cells, immune checkpoint blockade/deletion and the optimization of CAR-T cell anti-tumor functions (6). One promising avenue to enhance CAR-T cell function is optimizing the co-stimulatory signaling domains and/or incorporating the ability to secrete stimulant cytokines (such as IL-12, IL-18 or IL-15) (7–9). While these approaches are effective, they complicate CAR-T cell preparation and may introduce unforeseen systemic risks due to prolonged cytokine secretion. Another promising method to improve CAR-T cell efficacy is combination therapy, involving the integration of CAR-T cells with specific drugs or immunomodulators (10). Despite numerous studies exploring combination strategies to enhance the anti-solid tumor efficacy of CAR-T therapy, effective strategies that can address multiple solid tumors remain highly desirable but challenging due to the significant clinical demand and the inherent heterogeneity of solid tumors.

Lentinan (LTN), a polysaccharide extracted from *Lentinula edodes (L. edodes)*, has garnered attention due to its immunomodulatory, antiviral, and antitumor properties (11). The active component of LTN is β-(1→3)-D-glucan, with a branched structure consisting of β-(1→3)-linked glucose units and β-(1→6)-linked glucose branches distributed in a comb-like pattern (11, 12). Previous studies demonstrated that LTN exerts its antitumor effects primarily through immune modulation rather than direct cytotoxicity against cancer cells (11). Specifically, LTN has been shown to promote IFN-γ production while reducing IL-4 production by T cells *in vitro* (11, 13). However, the effects of LTN on CAR-T cells remain poorly understood.

Given LTN’s immunomodulatory properties, we hypothesized that its combination with CAR-T cells could enhance the activity of CAR-T cells within solid tumors. Therefore, we conducted a proof-of-concept study and confirmed that LTN can enhance the potency of CAR-T cells against multiple types of solid tumors by modulating CAR-T cell phenotypes. Our study reveals the role and mechanism of LTN in CAR-T cell functions and provides a novel combination therapeutic strategy for treating solid tumors.

## 2 Materials and methods

### 2.1 Cell line and constructs

Mouse melanoma cell line B16F10, mouse colorectal carcinoma cell lines MC38, and HEK293T cells were cultured in DMEM medium supplemented with 10% FBS (Gibco), 100 U/ml penicillin and 100 μg/ml streptomycin). To generate B16F10 and MC38 cell line stably expressing human EGFRvIII, B16F10 and MC38 cells were transduced with a lentiviral vector encoding human EGFRvIII, firefly luciferase and puromycin N-acetyltransferase (pLV-EGFRvIII-P2A-FLuc-T2A-puro) and selected on 7.5 μg/ml puromycin (Beyotime). To generate B16F10 and MC38 cell line stably expressing human CD19, the cells were transduced with a lentiviral vector encoding truncated human CD19, luciferase and puromycin N-acetyltransferase (pLV-hCD19t-P2A-FLuc-T2A-puro) and selected on 7.5 μg/ml puromycin (Beyotime). The expression of human EGFRvIII and CD19 was evaluated by flow cytometry (CytoFLEX S, Beckman Coulter) using an anti-EGFRvIII antibody (abcam, EPR28380-83) and APC anti-human CD19 antibody (Biolegend, clone: HIB19).

### 2.2 Animals

Six-to eight-week-old female C57BL/6J mice were used for all studies and maintained in individually ventilated cages under 12 h light/dark cycles in a specific pathogen-free animal facility. All animal studies were performed in accordance with the National Institutes of Health guidelines for the use and care of live animals and approved by the Institutional Animal Care and Use Committee of the Shenzhen Institutes of Advanced Technology, Chinese Academy of Sciences (SIAT-IACUC-241226-YYS-WXC-A2900).

### 2.3 Cloning and constructs

To generate murine CAR-T cells, a previously described CAR sequence targeting the human EGFRvIII and CD19 was used, which contains single-chain fragment (scFv) domain targeting EGFRvIII and CD19 (derived from antibody clone 139 and FMC63) (14, 15), a CD28 transmembrane domain, a CD28 costimulatory signal and a CD3ζ domain. based on the CAR sequence, a flag-tag was inserted between the signal peptide and the scFv domain. The plasmid MIGR1 was used as a retroviral vector (RV) backbone to construct pRV-EGFRvIII CAR and pRV-hCD19 CAR plasmids. The sequences were inserted in the MIGR1 plasmid between the EcoR I and Sal I restriction enzyme sites. All sequences were synthesized and subcloned by GENEWIZ.

### 2.4 Retrovirus preparation

Retroviral supernatants used for the transduction of murine T cells were prepared as previously described (16). In brief, retrovirus was produced by seeding 6-6.5 million HEK293T cells into 10 cm tissue culture dishes and transfecting the next day with 7.5 μg retroviral vector and 5 μg pCL-Eco plasmid that encodes gag/pol/env using polyethylenimine (Yeasen) at a 3:1 ratio. Supernatant containing the retrovirus was collected 48 h after transfection, and filtered with 0.45 μm filters. Retroviral supernatants were stored at 4°C or frozen at −80°C.

### 2.5 Murine T cell isolation and CAR-T cell production

Spleen T cells were isolated from 6-8-week-old C57BL/6J mice and mechanically disrupted. T cells were isolated using MojoSort™ Mouse CD3 T Cell Isolation Kit (Biolegend). The T cells were then activated for 2 days using plates coated with anti-mouse CD3 (5 μg/ml, Biolegend, clone: 145-2C11), soluble anti-mouse CD28 (2 μg/ml, Biolegend, clone: 37.51) monoclonal antibodies and 50 U/ml murine IL-2(10 ng/ml, Peprotech) in complete medium (RPMI-1640, Gibco) supplemented with 10% FBS (Gibco), 2 mM GlutaMAX, 50 μM β-mercaptoethanol, 100 U/ml penicillin and 100 μg/ml streptomycin). T cells were transduced with retroviral supernatants using retronectin-coated plates (Takara). 12 to 24 h after virus transduction, T cells were replaced with a new complete medium with 50 U/ml murine IL-2. On day 5, transduction efficiency was evaluated by surface staining of an APC anti-DYKDDDDK tag antibody (Biolegend, Clone: L5). T cells were then harvested and used for further assays.

### 2.6 Mouse T-cell cytotoxicity assay

The cytotoxicity assay was performed using luciferase-expressing target cells. Murine T cells were co-cultured with firefly luciferase-tagged target cells EGFRvIII^+^MC38 at indicated ratios for 24 hours, in a total volume of 200 μl in a 96-well plate. Luciferase expression and activity were used as indicators of target cell viability. The luminescent signal was detected by the POLARstar Omega multifunctional microplate reader (BMG LABTECH) after removal of cell culture supernatant and addition of D-Luciferin potassium salt substrate (Beyotime).

### 2.7 Enzyme−linked immunosorbent assay

Tumor lysates and supernatants from co-cultures of T cells and target cells were used for cytokine assays. Mouse IFN-γ and IL-2 ELISA kits (4A Biotech) were used to test the secretion of IFN-γ and IL-2 according to the manufacturer’s instructions. Duplicate wells were provided for each sample. The concentration of protein was calculated by measuring the optical density (OD) value of the absorbance at 450 nm.

### 2.8 Cell proliferation and viability assays

The B16F10 and MC38 cells were seeded in 96-well plates and treated with varying concentrations of LTN for different time periods, and then incubated with CCK-8 reagent (NCMBio) for 4 hours. Absorbance was measured at a wavelength of 450nm using a microplate reader (Thermo).

### 2.9 Quantitative real-time PCR

Total RNA of cells or tissues was isolated using the EasyPure RNA Kit (TransGen). cDNA was then synthesized with Hifair AdvanceFast 1st Strand cDNA Synthesis Kit (Yeasen) according to the manufacturer’s instructions. qRT-PCR was performed with TB Green Fast qPCR Mix (Takara) on a Bio-Rad CFX96 system (Bio-Rad), with reactions run in triplicate. Relative mRNA levels were normalized to the endogenous control gene *Gapdh* and determined using the comparative threshold cycle (Ct) method. Primer sequences are listed in **Supplementary Table 1**.

### 2.10 Adoptive transfer of murine CAR-T cells in murine tumor models

Female C57BL/6J mice (6–8 weeks old) were inoculated s.c. with 1×10^6^ EGFRvIII^+^MC38, EGFRvIII^+^B16F10, hCD19^+^MC38 or hCD19^+^B16F10 cells in 100 μl DPBS into the right flank. Mice were randomly assigned 7 days later, when tumors had grown to approximately 100 mm^3^. Then, the mice were then treated with 1×10^7^ T cells injected into the lateral tail vein.

In addition, mice in the CAR-T + LTN group were intraperitoneally injected with 1.25 mg/kg LTN (Solarbio) daily for 7 days. Tumor size and body weight were measured every two days. Mice were euthanized when the tumor volume reached 1500 mm^3^. On day 10 after infusion, some mice were euthanized and tumor tissues were collected and stored at -80°C for further analysis.

### 2.11 Phenotypic analysis of T cells

Tumors were collected and enzymatically digested by Collagenase I (Gibco) into single-cell suspensions. The single-cell suspensions were washed with PBS containing 1% FBS. Mouse TruStain FcX™ PLUS (BioLegend) was used for reducing non-specific immunofluorescent staining. For analysis of memory-type phenotypes and degranulation, the cells were stained with PE anti-mouse CD45 antibody (BioLegend, Clone:30-F11), FITC anti-mouse CD3 antibody (BioLegend, Clone:17A2), Brilliant Violet 510™ anti-mouse CD4 antibody (BioLegend, Clone: RM4-5), Alexa Fluor^®^ 700 anti-mouse CD8a antibody (BioLegend, Clone: 53-6.7), APC/Fire™ 750 anti-mouse CD62L antibody (BioLegend, Clone: MEL-14), APC anti-mouse CD44 antibody(BioLegend, Clone: IM7) and Brilliant Violet 421™ anti-mouse CD107a (LAMP-1) Antibody (BioLegend, Clone:1D4B). For analysis of the exhausted phenotype, cells were stained with PE anti-mouse CD45 antibody (BioLegend, Clone:30-F11), FITC anti-mouse CD3 antibody (BioLegend, Clone:17A2), Brilliant Violet 510™ anti-mouse CD4 antibody (BioLegend, Clone: RM4-5), Alexa Fluor^®^ 700 anti-mouse CD8a antibody (BioLegend, Clone: 53-6.7), APC/Fire™ 750 anti-mouse CD279 (PD-1) antibody (BioLegend, Clone: 29F.1A12), APC anti-mouse CD223 (LAG-3) antibody (BioLegend, Clone: C9B7W), Brilliant Violet 421™ anti-mouse CD366 (Tim-3) antibody (BioLegend, Clone: RMT3-23), FITC anti-mouse CD317 (BST2, PDCA-1) antibody (BioLegend, Clone: 927), APC/Fire™ 750 anti-human/mouse Granzyme B recombinant antibody (BioLegend, Clone: QA16A02), TCF1/TCF7 (C63D9) Rabbit mAb (Alexa Fluor^®^ 488 Conjugate, CST), Brilliant Violet 421™ anti-mouse CD127 (IL-7Rα) antibody (BioLegend, Clone: A7R34) and PE anti-mouse KLRG1 (MAFA) recombinant antibody (BioLegend, Clone: 2F1). After that, 7-AAD Viability Staining Solution (BioLegend) was added to stain the dead cells, and then the samples were detected and analyzed.

## 3 Results

### 3.1 LTN enhanced the antitumor activity of CAR-T cells *in vitro*

**Figure 1:**
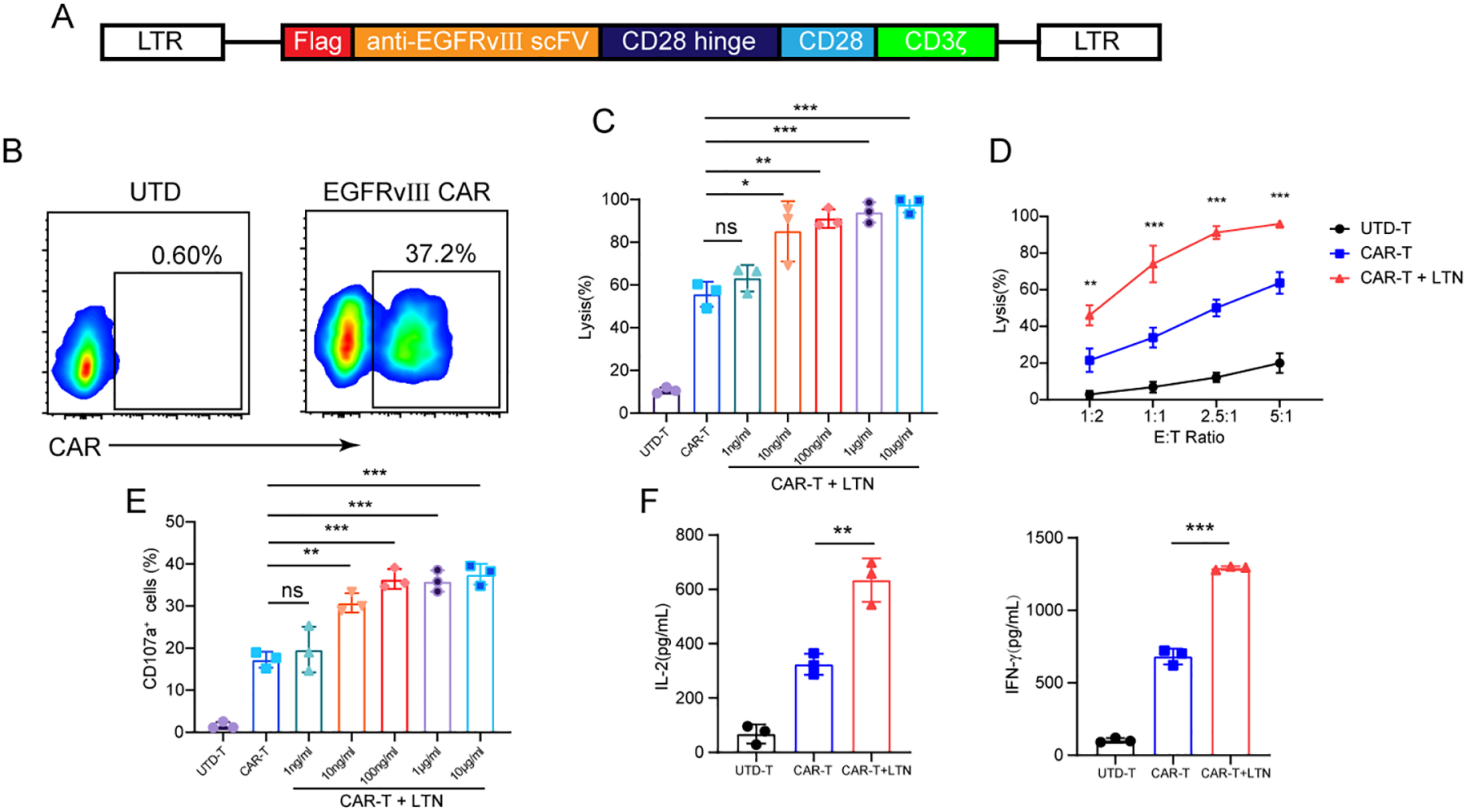
LTN enhanced the antitumor activity of CAR-T cells in vitro. **(A)** Schematic diagram of engineered murine CAR constructs. **(B)** The transfection efficiency of CAR-T cells was tested by flow cytometry. **(C)** Cytotoxicity assay using EGFRvIII^+^MC38 cells as target cells. CAR-T cells were co-cultured with target cells at various concentrations of LTN at the E/T ratio of 2:1 for 24 h **(D)** Cytotoxicity assay using EGFRvIII^+^MC38 cells as target cells. CAR-T cells were co-cultured with target cells at 10 ng/mL LTN at the indicated effector-to-target (E/T) ratios for 24 h **(E)** CD107a^+^ cells were detected using EGFRvIII^+^MC38 cells as target cells. CAR-T cells were co-cultured with target cells at various concentrations of LTN at the E/T ratio of 2:1 for 24 h **(F)** ELISA assay for IL-2 and IFN-γ secretion of engineered T cells when they were co-cultured with target cells at 10 ng/mL LTN at an E/T ratio of 2:1 for 24 hours. The results are representative of at least 3 independent experiments. *p < 0.05, **p < 0.01, ***p < 0.001, ns, not significant, (repeated-measures one-way ANOVA or Student t test). UTD-T, Untransduced - T.

To investigate the effect of LTN on CAR-T cell function, we first constructed a CAR targeting the mutant EGFRvIII (**Figure 1A**). Flow cytometry confirmed efficient CAR surface expression on mouse T cells (**Figure 1B**). We then co-cultured these CAR-T cells with EGFRvIII-overexpressing MC38 colon cancer cells and concurrently treated them with varying LTN concentrations. While literature reports direct cytotoxic effects of LTN on tumor cells (17, 18), no obvious toxicity was observed at any dose tested here (**Supplementary Figure S1A**). However, LTN at a concentration of 10 ng/ml significantly improve the tumor-killing activity of CAR-T cells (**Figure 1C**). While the antitumor effect increased with higher LTN concentrations, the response was not strictly concentration-dependent (**Figure 1C**). Specially, at 10 ng/ml LTN, the lytic ability of CAR-T cells against tumor cells gradually increased with escalating effector-to-target (E/T) ratios (**Figure 1D**). Moreover, LTN treatment resulted in an increased percentage of CD107a^+^ CAR-T cells (**Figure 1E**), indicating enhanced degranulation and cytotoxic potential. LTN treatment also upregulated the secretion of key cytokines, including IL-2 and IFN-γ, further demonstrating its role in boosting CAR-T cell-mediated anti-tumor responses (**Figure 1F**). These findings indicate that LTN can significantly improve the antitumor activity of CAR-T cells at relatively low concentrations *in vitro*.

### 3.2 LTN optimizes CAR-T cell differentiation *in vitro*

**Figure 2:**
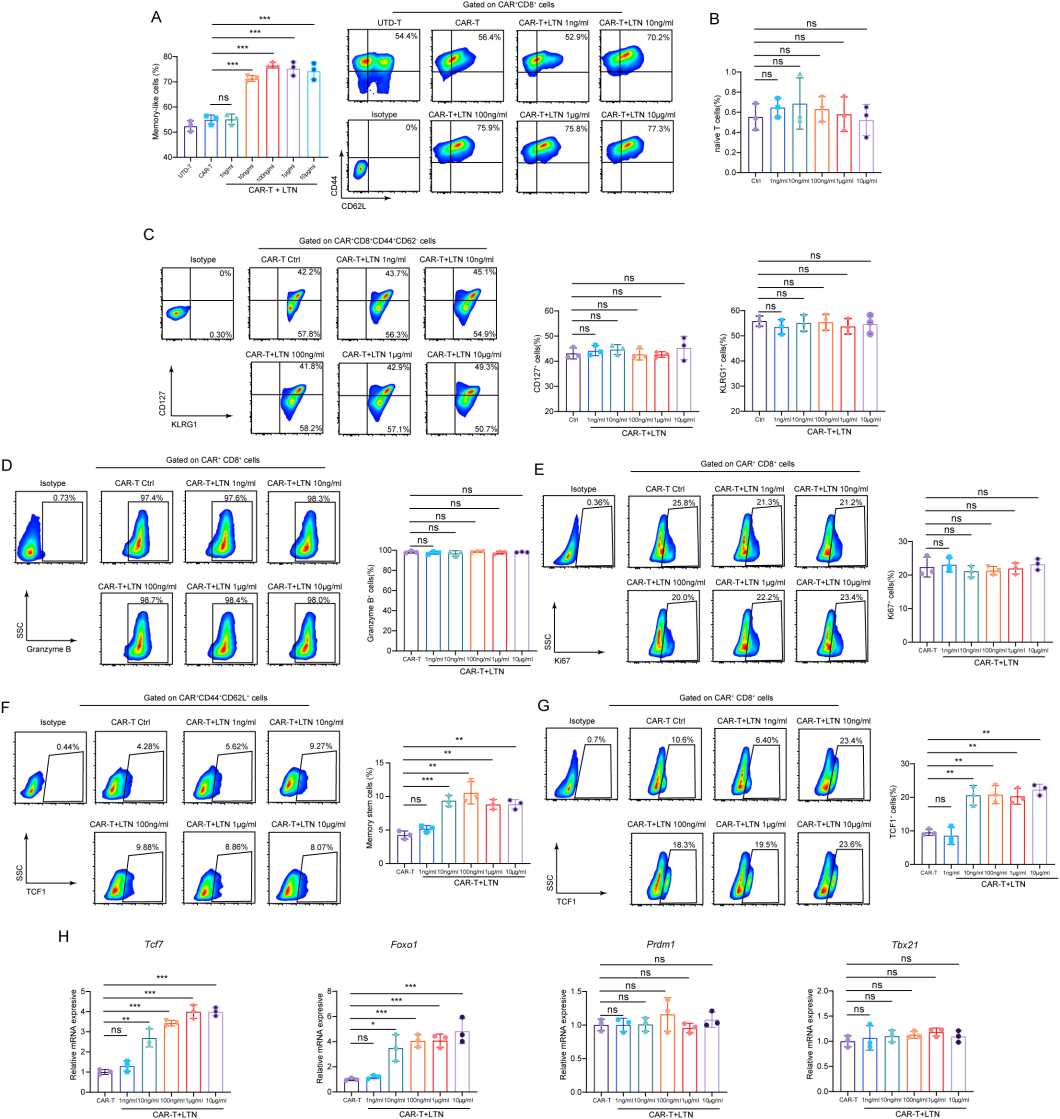
Characteristics of phenotypic differentiation of T cells after co-culture with target cells *in vitro*. **(A)** The percentages and representative flow cytometry plots of CD8^+^memory-like cells (CD44^+^CD62L^+^) were detected using EGFRvIII^+^MC38 cells as target cells. All the samples were gated on CAR^+^CD8^+^T cells. **(B)** The percentages naïve T (CD62L^+^CD44^-^) CAR-T cells were detected using EGFRvIII^+^MC38 cells as target cells. All the samples were gated on CAR^+^CD8^+^ cells. **(C)**The percentages and representative flow cytometry plots of Tem and Teff CAR-T cells were detected using EGFRvIII^+^MC38 cells as target cells. All the samples were gated on CAR^+^CD8^+^CD44^+^CD62L^-^ cells. **(D, E)** The percentages and representative flow cytometry plots of Granzyme B^+^ **(D)** and Ki67^+^ **(E)** T cells were detected using EGFRvIII^+^MC38 cells as target cells. All the samples were gated on CAR^+^CD8^+^T cells. **(F)** The percentages and representative flow cytometry plots of stem memory CAR-T cells were detected using EGFRvIII^+^MC38 cells as target cells. All the samples were gated on CAR^+^ CD8^+^CD44^+^CD62L^+^ cells. **(G)** The percentages and representative flow cytometry plots of TCF1^+^ T cells were detected using EGFRvIII^+^MC38 cells as target cells. All the samples were gated on CAR^+^CD8^+^T cells. **(H)** T cell memory differentiation-associated genes mRNA levels were analyzed by qPCR. The results are representative of at least 3 independent experiments. *p < 0.05, **p < 0.01, ***p < 0.001, ns, not significant, (repeated-measures one-way ANOVA or Student t test). UTD-T, Untransduced - T.

Central memory T cells are characterized by self-renewal capabilities and long-term survival, and their reactivation by tumor antigens leads to robust tumor cell killing (19). Given the importance of memory T cells for sustained anti-tumor immunity, we next assessed the effect of LTN on the differentiation of CAR-T cells into a central memory (Tcm, CD44^+^CD62L^+^) phenotype. After 48 hours of co-culture with EGFRvIII^+^MC38 target cells, we analyzed the phenotypes of CAR^+^CD8^+^T cells by flow cytometry. Our results showed that LTN treatment significantly increased the proportion of central memory CAR-T cells, with a marked effect observed at 10 ng/mL LTN (**Figure 2A**; **Supplementary Figure S1B**). Notably, this increase was not concentration-dependent, as the proportion of Tcm cells plateaued within the concentration range tested. This suggests that LTN’s effect on CAR-T cell differentiation reaches an optimal threshold at a low dose. We further analyzed the impact of LTN on CAR-T cell subsets, including naïve T, effector memory T (Tem), effector T cells (Teff) and stem cell-like memory T cells (Tscm). LTN had no significant effect on the differentiation of naïve T, Tem and Teff cells (**Figures 2B, C**). Similarly, it did not alter CAR-T cell proliferation (Ki67^+^) or granzyme B expression levels (**Figures 2D, E**), potentially due to universally high baseline granzyme B expression in these CAR-T cells. However, LTN significantly promoted differentiation toward a Tscm phenotype (**Figure 2F**) and enhanced stemness of CAR-T cells (**Figure 2G**). To explore how LTN promotes memory differentiation in CAR-T cells, we assessed transcription of T cell memory-related genes. LTN increased mRNA levels of the memory reprograming regulators *Tcf7* (encoding TCF1) and *Foxo1*, without altering effector-associated genes (*Prdm1* and *Tbx21)* (**Figure 2H**). This selective upregulation implicates the TCF1/FOXO1 pathway in LTN-mediated induction of a memory phenotype.

**Figure 3:**
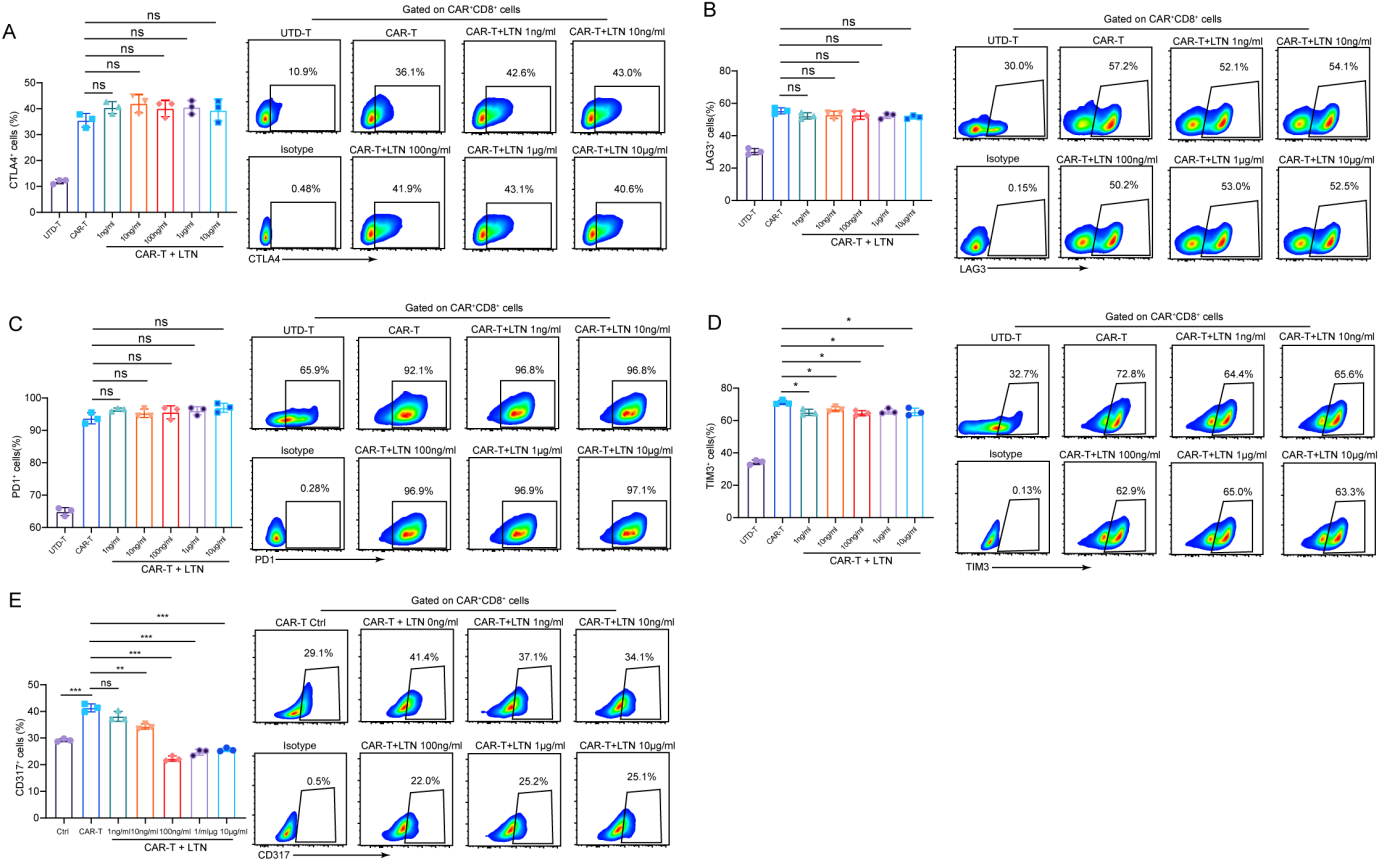
Analysis of CAR-T cell functional markers **(A-E)** The percentages and representative flow cytometry plots of CD8^+^CTLA4^+^ **(A)**, LAG3^+^ **(B)**, PD1^+^ **(C)**, TIM3^+^ **(D)** and CD317^+^ **(E)** T cells were detected using EGFRvIII^+^MC38 cells as target cells. All the samples were gated on CAR^+^CD8^+^T cells. The results are representative of at least 3 independent experiments. *p < 0.05, **p < 0.01, ***p < 0.001, ns, not significant, (repeated-measures one-way ANOVA or Student t test).

CAR-T cell exhaustion is a major challenge, especially in the treatment of solid tumors, where CAR-T cells often exhibit a functional decline over time (20). To explore whether LTN could mitigate CAR-T cell exhaustion, we analyzed the expression of exhaustion markers such as CTLA-4, LAG-3, PD-1, and TIM-3. While LTN treatment did not change the expression of CTLA-4, LAG-3 and PD-1 (**Figures 3A-C**) nor the co-expression patterns of exhaustion markers (TIM3^+^LAG3^+^, TIM3^+^PD1^+^ and TIM3^+^CTLA4^+^) (**Supplementary Figure S2A**), it significantly reduced the expression of TIM-3 (**Figure 3D**) and, more notably, CD317 (**Figure 3E**). CD317 is a newly identified exhausted T cell marker in HCC and had been shown to exert immunosuppressive function in NK cells (21, 22). This reduction in TIM-3 and CD317 expression suggests that LTN may alleviate CAR-T cell exhaustion, further promoting their anti-tumor activity. Taken together, our findings provide compelling evidence that LTN significantly enhances the anti-tumor activity of CAR-T cells *in vitro* by promoting the differentiation of CAR-T cells into central memory phenotypes and by modulating key markers of exhaustion.

### 3.3 LTN enhances the antitumor activity of CAR-T cells *in vivo*

**Figure 4:**
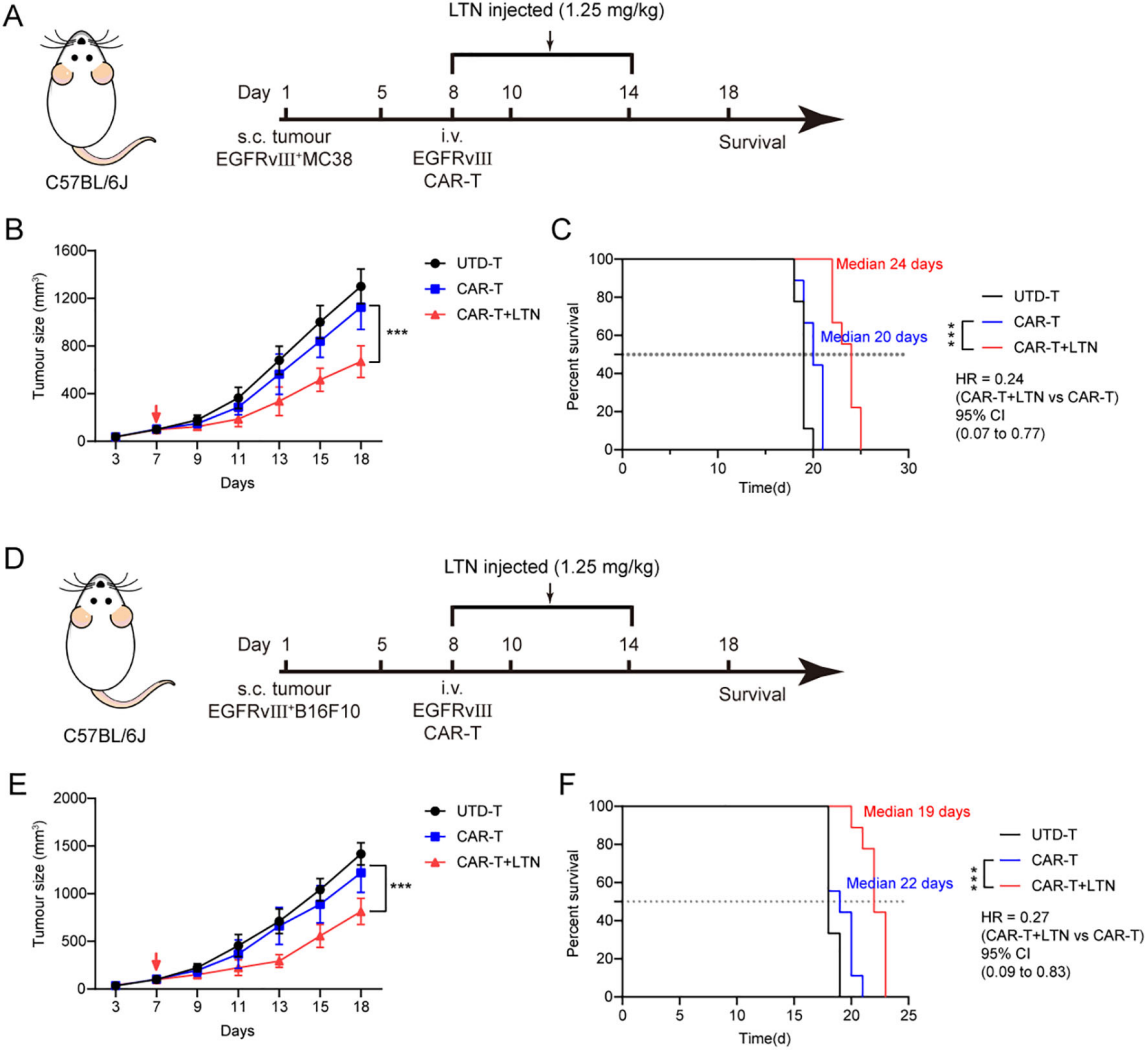
LTN enhanced the antitumor activity of CAR-T cells in vivo. **(A)** Treatment schedule for subcutaneous murine EGFRvIII^+^MC38 colorectal carcinoma cells using engineered T cells. **(B)** The changes in tumor volume over time after tumor cell implantation, n = 9 mice/group. The red arrow indicates the time point of intravenous T cell injection. **(C)** The Kaplan-Meier survival curves after treatment for EGFRvIII^+^MC38 tumor-bearing mice. n = 9 mice/group. **(D)** Treatment schedule for subcutaneous murine EGFRvIII^+^B16F10 melanoma cells using engineered T cells. **(E)** The changes in tumor volume over time after tumor cell implantation, n = 9 mice/group. The red arrow indicates the time point of intravenous T cell injection. **(F)** The Kaplan-Meier survival curves after treatment for EGFRvIII^+^B16F10 tumor-bearing mice. n = 9 mice/group. Tumor sizes between treatment groups were compared using two-way ANOVA, and survival curves were compared using the log-rank test. ***p < 0.001. HR, hazard ratios. CI, confidence interval. UTD-T, Untransduced - T.

To evaluate the potential of LTN in boosting the anti-tumor activity of CAR-T cells in solid tumors, we used a syngeneic EGFRvIII^+^MC38 colon cancer model in immunocompetent mice. Following tumor cell implantation, engineered murine T cells were intravenously administered to the tumor-bearing mice (**Figure 4A**). Tumor volume and body weight were monitored over time. Our findings demonstrated that LTN could significantly improve the tumor-inhibitory effects of CAR-T cells and prolong the survival of treated mice (**Figures 4B, C**). We also investigated whether LTN alone could exhibit comparable anti-tumor effects to CAR-T cells. Although LTN alone could inhibit tumor growth, CAR-T cells combined with LTN produced the most potent tumor-suppressing effects (**Supplementary Figure S3A, B**). Furthermore, in a separate experiment using the EGFRvIII^+^B16F10 melanoma model (**Figure 4D**), LTN similarly enhanced the anti-tumor effects of CAR-T cells (**Figures 4E, F**). These results indicated that LTN could effectively enhance the anti-tumor activity of CAR-T cells *in vivo* across different solid tumor models.

### 3.4 LTN synergistically enhances CAR-T cell efficacy and promotes pro-inflammatory TAM polarization in tumors

**Figure 5:**
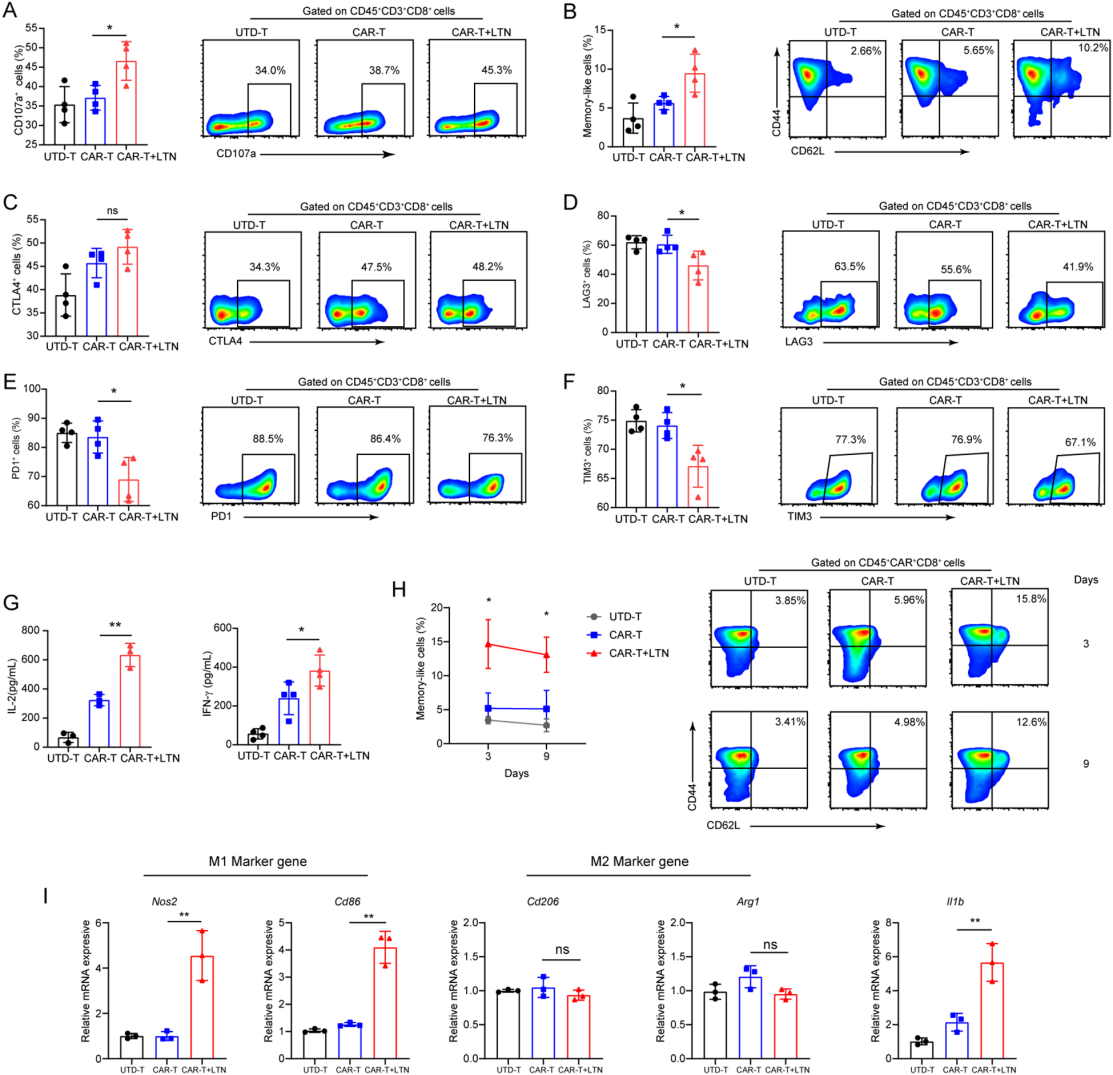
LTN can remodel the tumor microenvironment and change the phenotype of T cells. **(A–F)** The percentages and representative flow cytometry plots of tumor-infiltrating CD3^+^CD8^+^CD107a^+^ **(A)**, central memory (CD44^+^CD62L^+^) CD8^+^T cells **(B)**, CTLA4^+^ **(C)**, LAG3^+^ **(D)**, PD1^+^ **(E)** and TIM3^+^ **(F)** T cells were detected. All the samples were gated on CD45^+^CD3^+^CD8^+^T cells. **(G)** The quantities of IL-2 and IFN-γ in tumor lysates were evaluated by ELISA. **(H)** The percentages and representative flow cytometry plots of tumor-infiltrating Tcm cells were detected at different time points after CAR-T infusion. **(I)** M1 (*Nos2* and *Cd86*), M2 (*Arg1* and *Cd206*) marker genes and pro-inflammatory (*Il1b*) gene of macrophages were analyzed by qPCR, *p < 0.05, **p < 0.01, ns, not significant, (repeated-measures one-way ANOVA or Student t test). UTD-T, Untransduced - T.

Given the observed enhancement of CAR-T cell-mediated tumor suppression by LTN, we next explored whether LTN could affect the activation and phenotype of tumor-infiltrating T cells. Flow cytometric analysis showed that LTN, when combined with CAR-T cells, significantly increased the proportion of CD8^+^CD107a^+^T cells within tumors (**Figure 5A**), as well as the proportion of central memory cells (**Figure 5B**). Critically, while CTLA-4 expression did not significantly differ between groups (**Figure 5C**), CAR-T+LTN-treated mice exhibited markedly reduced expression of the inhibitory receptors PD-1, TIM-3, and LAG-3 in CAR-T cells (**Figures 5D–F**), along with significantly diminished co-expression frequencies of TIM-3^+^PD-1^+^, TIM-3^+^LAG-3^+^, and TIM-3^+^CTLA-4^+^ subsets (**Supplementary Figures S2B**). Similarly, increased levels of IL-2 and IFN-γ were observed in the tumors of CAR-T+LTN-treated mice (**Figure 5G**). We also assessed the durability of tumor-infiltrating CAR-T cells, finding a significant proportion of central memory-like T cells persisted within tumors at day 9 post-treatment (**Figure 5H**). These data indicate that LTN improves CAR-T cell anti-tumor efficacy by encouraging a favorable phenotype, durability, and activity. Given prior reports that LTN activates tumor-associated macrophages (TAMs) and induces inflammatory cytokine secretion in murine models (23), we also investigated its impact on TAM polarization. qPCR analysis revealed LTN increased transcription of M1-associated genes (*Nos2, Cd86*) and *Il1b* within tumors, while leaving M2-associated gene expression unaltered (**Figure 5I**). This shift toward a pro-inflammatory TAM phenotype suggests LTN also indirectly enhances CAR-T cell function by modulating the tumor microenvironment. Collectively, our findings demonstrate that LTN not only directly potentiates CAR-T cell anti-tumor activity but also reprograms TAMs toward an immunostimulatory state, thereby augmenting overall anti-tumor immunity.

### 3.5 LTN enhances the anti-tumor activity of CAR-T cells against multiple targets

**Figure 6:**
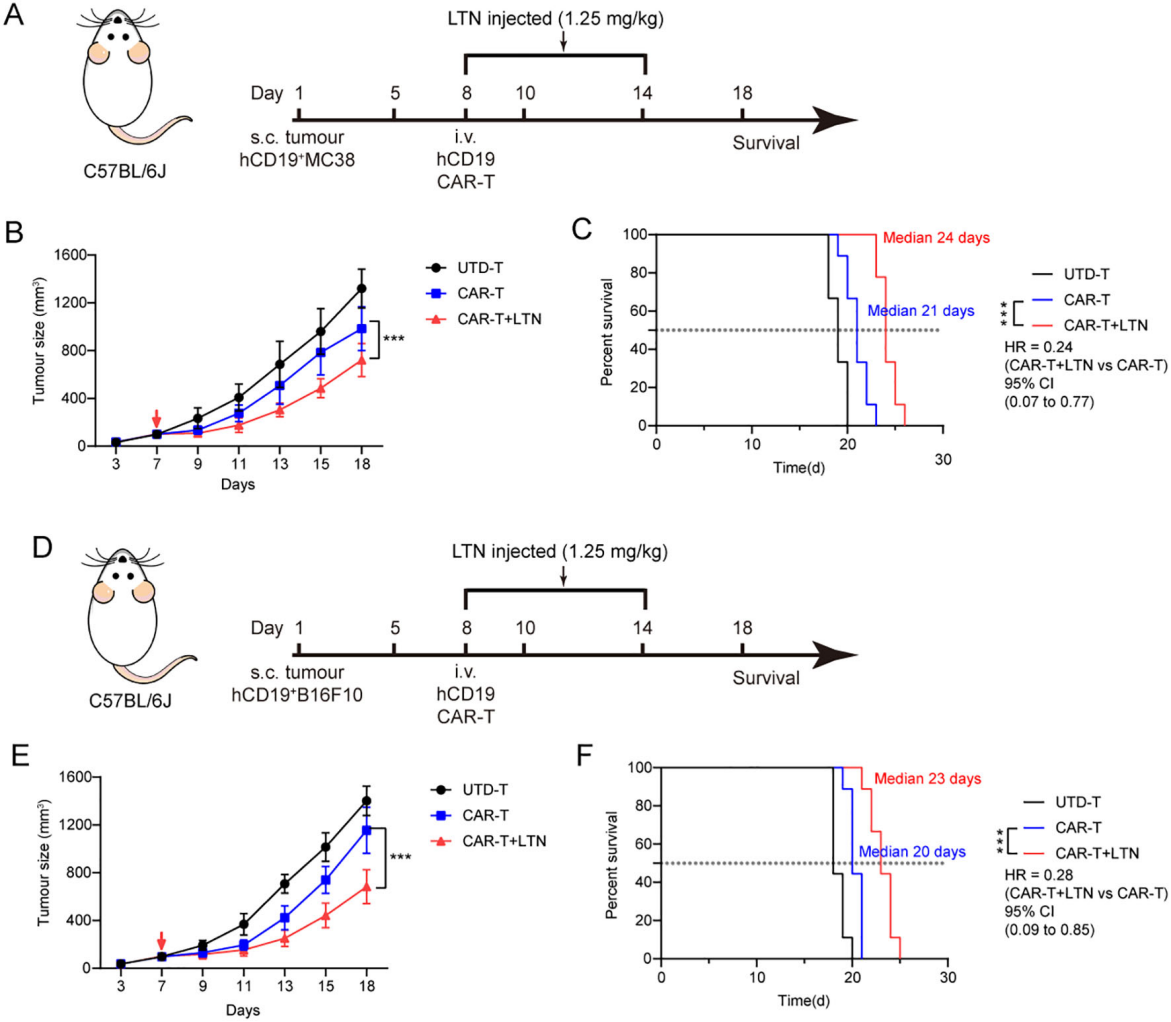
LTN can enhance the anti-tumor activity of CAR-T cells in more targets. **(A)** Treatment schedule for subcutaneous murine hCD19^+^MC38 colorectal carcinoma cells using engineered T cells. **(B)** The changes in tumor volume over time after tumor cell implantation, n = 9 mice/group. The red arrow indicates the time point of intravenous T cell injection. **(C)** The Kaplan-Meier survival curves after treatment for hCD19^+^MC38 tumor-bearing mice. n = 9 mice/group. **(D)** Treatment schedule for subcutaneous murine hCD19^+^B16F10 melanoma cells using engineered T cells. **(E)** The changes in tumor volume over time after tumor cell implantation, n = 9 mice/group. The red arrow indicates the time point of intravenous T cell injection. **(F)** The Kaplan-Meier survival curves after treatment for hCD19^+^B16F10 tumor-bearing mice. n = 9 mice/group. Tumor sizes between treatment groups were compared using two-way ANOVA, and survival curves were compared using the log-rank test. ***p < 0.001. HR, hazard ratios. CI, confidence interval. UTD-T, Untransduced - T.

To determine whether LTN could enhance CAR-T cell activity against other tumor targets, we generated human-derived CD19-expressing MC38 and B16F10 cells (hCD19^+^MC38 and hCD19^+^B16F10) as alternative tumor targets of CAR-T therapy. We then established a syngeneic hCD19^+^MC38 colon cancer model in immunocompetent mice (**Figure 6A**). As expected, LTN treatment significantly improved the tumor-suppressive activity of CAR-T cells and prolonged the survival of treated mice (**Figures 6B, C**). Similar results were observed in the hCD19^+^B16F10 melanoma model (**Figures 6D–F**). These results indicate that the enhancement of CAR-T cell function by LTN is not restricted to a certain tumor target or cancer type. Instead, LTN has broad applicability in potentiating the efficacy of CAR-T therapy.

## 4 Discussion

CAR-T cell therapy has emerged as a promising and effective treatment modality for hematologic malignancies, demonstrating remarkable therapeutic responses. However, its efficacy in solid tumors has been hindered by multiple challenges, including immune suppression within the tumor microenvironment (TME). These factors often result in CAR-T cell dysfunction, exhaustion, and poor persistence *in vivo* (3, 4, 6, 24). To address these limitations, several strategies have been developed, including armed CAR-T cells that release cytokines, immune-regulatory ligands, or therapeutic antibodies, arming to enhance the efficacy of these cells in hostile TME (7–9, 25). Herein we report that LTN can effectively enhance CAR-T cell efficacy in the treatment of solid tumors. Our *in vitro* findings reveal that it not only boosts the cytotoxicity and cytokine secretion of CAR-T cells but also promotes the differentiation of a central memory-like phenotype, preserving their stemness characteristics. *In vivo*, LTN effectively promoted the therapeutic effect of CAR-T cells in melanoma and colon cancer tumor models, independent of tumor type or target antigen. This preclinical evidence supports the potential of LTN as a viable adjunct to CAR-T cell therapy in solid tumors.

LTN, derived from *Lentinus edodes* (*L. edodes*), is a biologically active compound known for its diverse biological activities, including immunomodulatory, antibacterial, antiviral, anti-inflammatory and antitumor properties (11, 26). LTN has been extensively studied, and is approved for use in adjuvant cancer treatments in countries such as China and Japan (27). LTN’s ability to modulate immune responses is of particular interest in enhancing the effectiveness of T cell-based therapies. In T cell biology, activated resting naïve T cells can differentiate into Teff cells and memory T cells. While Teff cells are potent killers that produce cytotoxic molecules and effector cytokines, their expansion and persistence *in vivo* are limited (28). In contrast, Tcm cells exhibit stem cell-like properties, allowing for long-term survival and rapid re-expansion upon antigen re-exposure (29, 30). The long-term success of CAR-T therapy depends on the persistence and expansion of CAR-T cells after infusion, which is crucial for controlling tumor growth and recurrence (31–33). CAR-T cells derived from Tcm subsets display superior anti-tumor efficacy and prolonged persistence compared to those produced from Tem (34, 35). In solid tumors, CAR-T therapy faces challenges in ensuring the longevity of CAR-T cells within the tumor, significantly limiting their anti-tumor efficacy. Given the multifaceted advantages of memory T cells, various strategies, such as supplementation with IL-7 and IL-15, have been developed to generate CAR-T cells with memory-like characteristics, improving CAR-T cell expansion and persistence (30, 36, 37). In addition, overexpression of FOXO1 or manipulation of T cell metabolism by targeting isocitrate dehydrogenase 2 (IDH2) has been shown to facilitate the generation of memory-like CAR-T cells and improve their anti-tumor activity *in vivo* (38–40). Our study demonstrated that LTN promotes the differentiation of CAR-T cells into a memory-like phenotype without compromising their anti-tumor function, offering a novel approach for enhancing CAR-T therapy. While LTN has previously been shown to inhibit tumor growth through various immune mechanisms, including IFN-γ production by tumor-infiltrating T cells (41), our findings suggest that the differentiation of CAR-T cells into a central memory-like phenotype may be a key mechanism underlying LTN’s ability to enhance CAR-T cell efficacy. However, further studies are needed to fully elucidate the specific molecular mechanisms by which LTN influences CAR-T cell differentiation.

A major challenge in CAR-T cell therapy for solid tumors is T cell exhaustion following tumor infiltration, characterized by upregulation of inhibitory receptors (e.g., PD-1, CTLA-4, LAG-3, TIM-3) and diminished proliferative capacity and antitumor activity. Mitigating CAR-T cell exhaustion to sustain their effector function is therefore critical for improving solid tumor treatment outcomes (20, 42). While our *in vitro* results show that LTN reduces TIM-3 expression without affecting other established exhaustion markers (PD-1, CTLA-4, and LAG-3), its pronounced suppression of CD317—a newly identified T cell exhaustion marker (21, 22)—suggests it may target a distinct exhaustion pathway. *In vivo*, LTN more robustly inhibited CAR-T cell exhaustion. Beyond TIM-3, LTN significantly downregulated additional immune checkpoints (including PD-1 and LAG-3) and reduced the frequency of co-expressing populations (e.g., TIM-3^+^PD-1^+^ cells). This phenotypic divergence between *in vitro* and *in vivo* settings may result from LTN-driven reprogramming of the tumor microenvironment (TME), particularly via TAM polarization. The immunosuppressive TME remains a major barrier to CAR-T efficacy in solid tumors, where M2-polarized TAMs are key immunosuppressive mediators (43). We demonstrate that combining LTN with CAR-T therapy promotes TAM repolarization toward the M1 phenotype and elevates pro-inflammatory cytokine expression within tumors. This TME reprogramming likely enhances anti-tumor efficacy by attenuating CAR-T cell exhaustion and potentiating endogenous anti-tumor immunity, thereby initiating a self-reinforcing synergistic loop. Therefore, LTN integration represents a promising strategy to augment CAR-T therapy for solid tumors.

Interestingly, our results reveal that LTN does not follow a dose-dependent response in CAR-T cell enhancement, contrasting with previous studies showing an inverted U-shaped dose-response for tumor growth inhibition (41). This could be attributed to differential phenotypic changes in CAR-T cells compared to unmodified T cells, highlighting that LTN may exert its effects through distinct mechanisms in engineered T cells. While LTN stimulates anti-tumor immunity, tumor size in mice receiving LTN combined with unmodified T cells did not differ significantly from those treated with LTN alone. However, when LTN is combined with CAR-T cells, a stronger tumor inhibition effect is observed, suggesting that LTN may enhance the anti-tumor activity of CAR-T cells through special mechanisms. Notably, LTN’s enhancement of CAR-T cells was not restricted by tumor type or target, as demonstrated in both melanoma and colon cancer models. This broad applicability increases the potential of LTN as a universal adjunct to CAR-T therapies across various solid tumors. While the results obtained from preclinical mouse models are promising, further studies in human CAR-T cells and tumor models are necessary to validate these findings and provide additional data for clinical translation.

In summary, our study introduced a novel combination strategy of CAR-T cell therapy by harnessing the immunomodulatory effects of LTN. LTN effectively improves the tumor-killing ability and cytokine production of CAR-T cells, particularly by promoting the differentiation of central memory-like CAR-T cells. The ability of LTN to improve CAR-T cell efficacy across various tumor types and targets provides a compelling foundation for future clinical investigations, offering a new avenue for improving the success of CAR-T cell therapy in solid tumors.

## Data Availability

The raw data supporting the conclusions of this article will be made available by the authors, without undue reservation.
