# Supplementary material for: Lentinan enhances CAR-T cell potency in solid tumors by optimizing T cell differentiation
Source: Front Immunol. 2025 Jul 18;16:1605488. doi: 10.3389/fimmu.2025.1605488 (PMC12313588; doi:10.3389/fimmu.2025.1605488)
Supplement: Supplementary file 1 [file DataSheet1.docx]

Supplementary Material

**Lentinan enhances the efficacy of CAR-T cell therapy against solid tumors**

Xiangyun Niu, *et al.*

*Corresponding author. Guizhong Zhang, gz.zhang@siat.ac.cn

Zhiming Xu, zm.xu@siat.ac.cn

**This PDF file includes:**

1. **Supplementary Figures**

Figures S1

Figures S2

Figures S3

1. **Supplementary Table 1**

Table 1

# Supplementary Figures

.
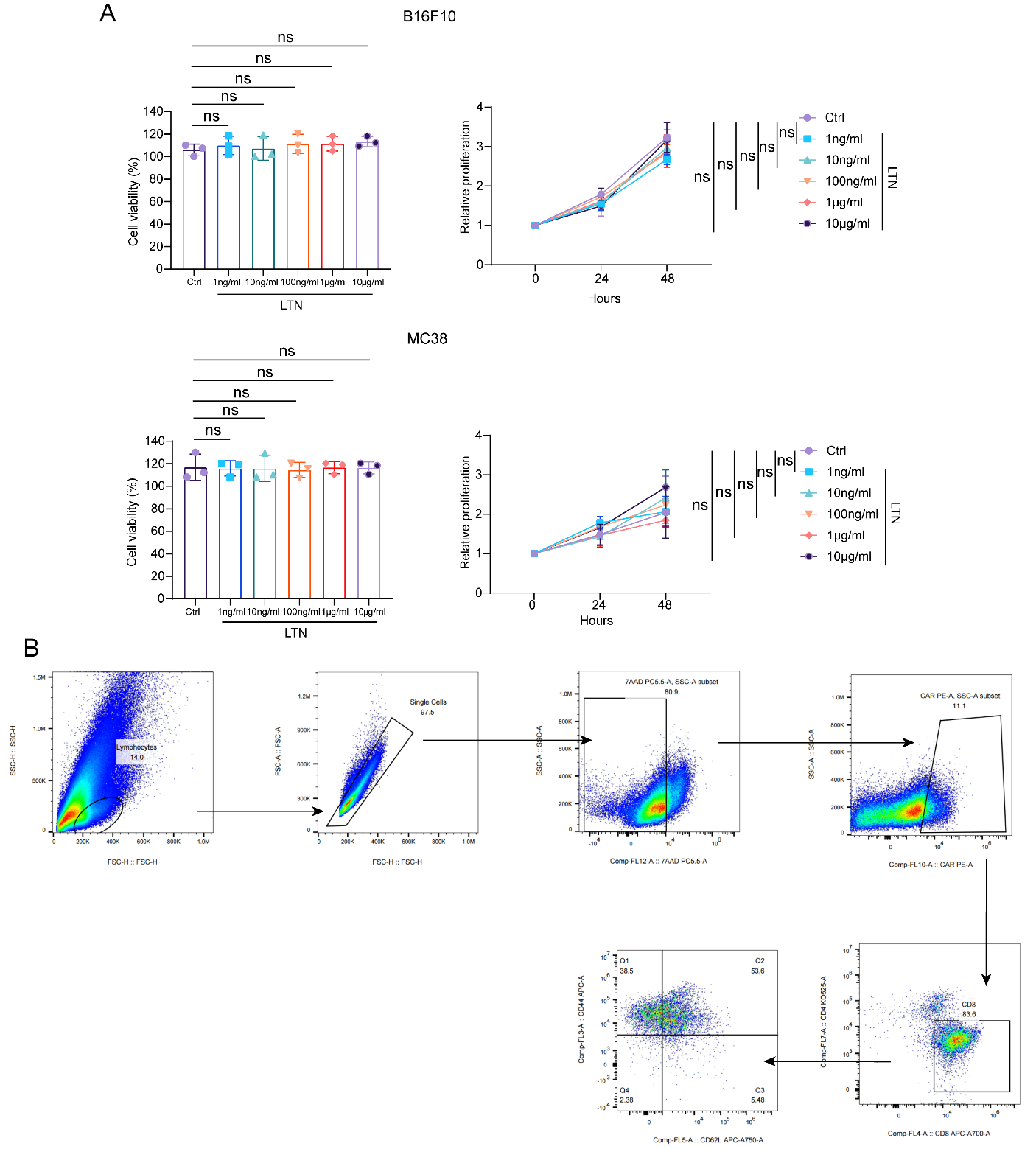


**Supplementary Figure S1. Tumor cell activity detection and memory-like T cell gate strategy.**

(A) Tumor cell B16F10 and MC38 cell viability and proliferation assays.

(B) The flow cytometry experiment gating strategy for CD44 and CD62L populations.

The results are representative of at least 3 independent experiments. ns, not significant, (repeated-measures one-way ANOVA or Student t test).


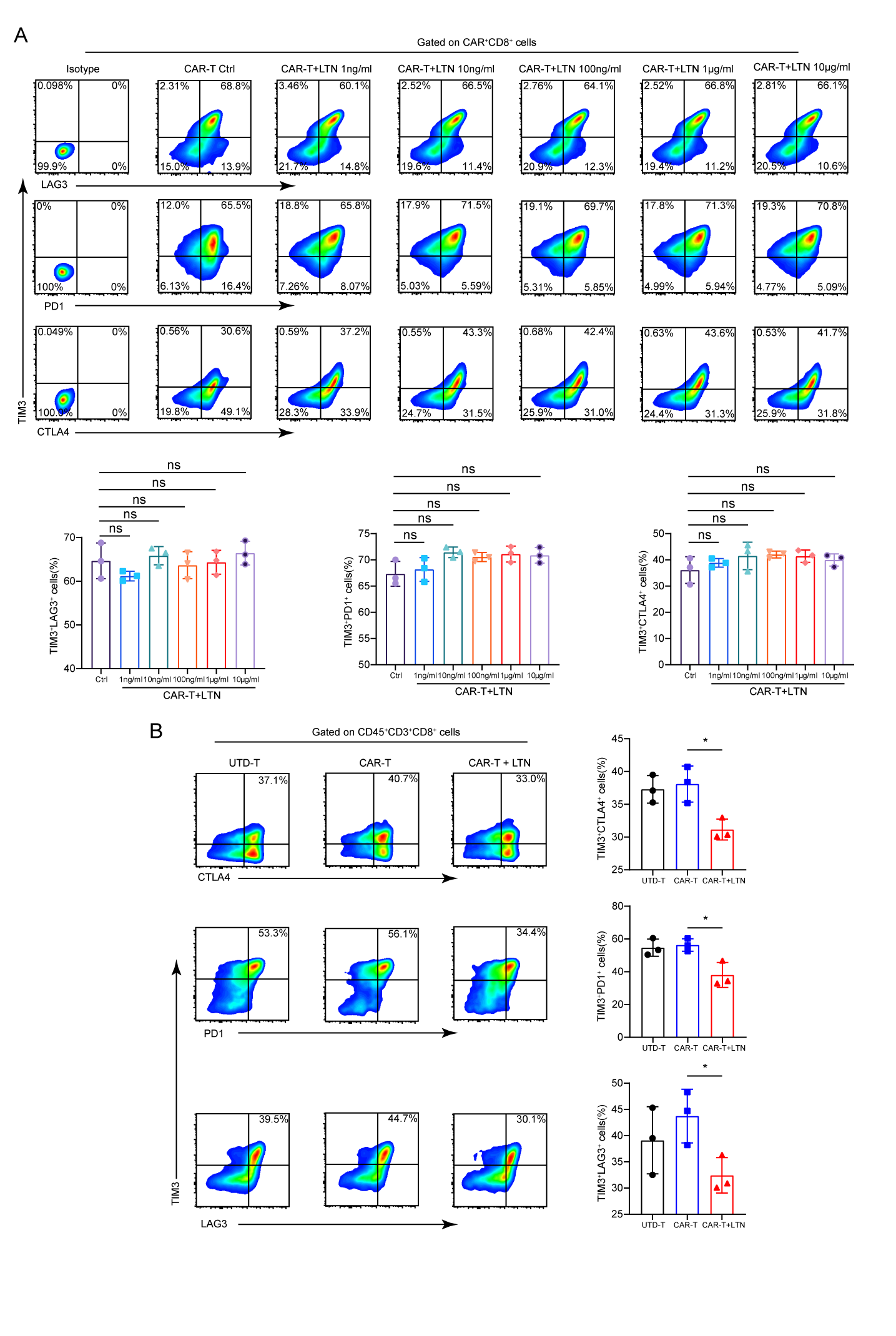


**Supplementary Figure S2. Analysis of co-expression patterns of T cell exhaustion markers.**

(A)The percentages and representative flow cytometry plots of co-expression patterns of exhaustion markers were detected using EGFRvⅢ^+^MC38 cells as target cells. All the samples were gated on CAR^+^CD8^+^T cells.

(B) The percentages and representative flow cytometry plots of tumor-infiltrating co-expression patterns of exhaustion markers were detected. All the samples were gated on CD45^+^CD3^+^CD8^+^T cells.

The results are representative of at least 3 independent experiments. **p* < 0.05. ns, not significant, (repeated-measures one-way ANOVA or Student t test).

**
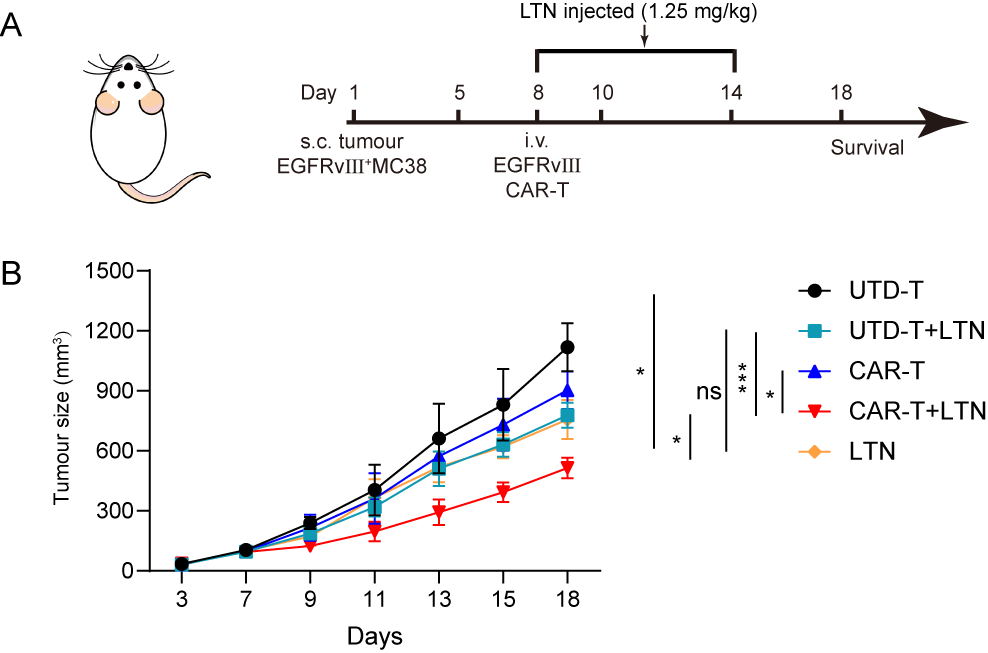
**

**Supplementary Figure S3.** **Therapeutic effect of LTN in combination with engineered T cells.**

(A) Treatment schedule for subcutaneous murine EGFRvⅢ^+^MC38 colorectal carcinoma cells using engineered T cells.

(B) The changes in tumor volume over time after tumor cell implantation, n = 4 mice/group. The red arrow indicates the time point of intravenous T cell injection.

Tumor sizes between treatment groups were compared using two-way ANOVA, **p* < 0.05, ****p* < 0.001, ns, not significant.

# Supplementary Table

**Table 1. Primer sequences for qPCR**

| Name | Forward (5'-3') | Reverse (5'-3') |
| --- | --- | --- |
| *Tcf7* | CCCTCAATGCGTTCATGCTT | TCATAGTACTTGGCCTGCTCT |
| *Foxo1* | TCAATTCGCCACAATCTGTCC | GTGATTTTCCGCTCTTGCCTC |
| *Prdm1* | CTTTCAAGTGCCAGACCTGCAA | CTCATGTGGCTTCTCTCCTGT |
| *Tbx21* | GTATCCTGTTCCCAGCCGTTT | CCGCTTCATAACTGTGTTCCC |
| *Nos2* | ACGCTTCACTTCCAATGCAAC | TGCGGCTGGACTTTTCACTC |
| *Cd86* | CGTGCCCATTTACAAAGGCTC | ATCAAGTTTCTCTGTGCCCAA |
| *Cd206* | ACTCTTTGGAATCAAGGGCACA | ATCTGCTCCACAATCCCGAAC |
| *Arg1* | AGAAATTTACAAGACAGGGCTC | CTTATGGTTACCCTCCCGTT |
| *Il1b* | CAGCTTCAAATCTCGCAGCAG | AAGAAGGTGCTCATGTCCTCA |
| *Gapdh* | AGAGTGTTTCCTCGTCCCGTA | ACAATCTCCACTTTGCCACTG |
